# The Effect of *Beauveria brongniartii* and its Secondary Metabolites on the Detoxification Enzymes of the Pine Caterpillar, *Dendrolimus tabulaeformis*

**DOI:** 10.1673/031.013.4401

**Published:** 2013-05-21

**Authors:** Jinhua Fan, Yingping Xie, Jiaoliang Xue, Rui Liu

**Affiliations:** The School of Life Science, Shanxi University, Taiyuan 030006, China

**Keywords:** entomopathogenic fungi, esterase, glutathione *S*-transferase, immune reaction, toxic

## Abstract

The mortality of pine caterpillar, *Dendrolimus tabulaeformis* Tsai et Liu (Lepidoptera: Lasiocampidae), larvae treated with *Beauveria brongniartii* (Saccardo) Petch (Hypocreales: Clavicipitaceae) conidia and cell-free culture supernatants enriched for the secondary metabolites of the fungus was investigated. In addition, the effects of the treatments on the activities of two insect-related defense response proteins, glutathione *S*-transferase (GST) and esterase (EST), were measured over time. Bioassays were performed using a range of fungal spore (6 × 10^5^ through 6 × 10^7^ spores/mL) and supernatant extract concentrations (5.5–550 µg/mL). The results showed that the mortalities of *D. tabulaeformis* larvae were closely related to the concentration of the conidia and the metabolites of *B. brongniartii*. The differences among the treatments all reached a significant level. The activities of the two detoxifying enzymes, GST and EST, in the larvae increased simultaneously post-treatment. After infection with the conidial suspensions, the highest GST activity appeared at 3 days, and the activities of the caterpillars infected with 6 × 10^6^ spores/mL and 6 × 10^7^ spores/mL were significantly higher than in the control. Using α-naphthyl, the highest activity of EST also appeared at 3 days, and the differences for the three different concentrations were significant. A similar trend of change in the EST activity was observed using β-naphthyl. After treatment with the secondary metabolite solution, the highest GST activity appeared at 6 hr, and significant differences were found both for the different durations (2, 4, 6, 12, 24, and 48 hr) and in the three concentration groups. When using α-naphthyl, the EST activity peak appeared at 24 hr, and the differences were significant among the durations of 2, 4, 6, 12, 24, and 48 hr. The effect of the concentration of the secondary metabolite solution notably induced the EST activity in the insects, and a similar result was obtained using β-naphthyl. The data suggest that *B. brongniartii* produces secondary metabolites that disable the immune mechanisms of *D. tabulaeformis*, allowing the fungus to overcome and then kill its host. It was concluded that both the conidial suspensions and the metabolites of *B. brongniartii* were toxic to *D. tabulaeformis* larvae.

## Introduction

The pine caterpillar, genus *Dendrolimus* (Lepidoptera: Lasiocampidae), is one group of the principal pests in coniferous forests worldwide. There are 27 species in China, and, according to the report from the National Forestry Department of China, areas of damage cover more than 2 million hm^2^ each year (Hou 1987). Since the 1970s, entomopathogenic fungi *Beauveria bassiana* and *Beauveria brongniartii* (Saccardo) Petch (Hypocreales: Clavicipitaceae) have been incorporated into an integrated pest management program to control the pine caterpillar (Wang et al. 2010).

The infection mechanism of *Beauveria* in the host insects has been reported (Roberts et al. 2004; Wang et al. 2004; Thomas et al. 2007). The primary route of host invasion is through the external integument via the attachment of the conidia to the cuticle, germination, followed by penetration into the cuticle. Once in the hemocoel, the mycelium ramifies throughout the host, forming yeast-like hyphal bodies or blastospores. Host death is often due to a combination of the action of a fungal toxin, the physical obstruction of blood circulation, nutrient depletion, and the invasion of organs.

During the attack process, the host immune system attempts to resist the fungus, and some detoxification enzymes inside the insect play a part in protecting the insects from the negative impact of the pathogens and their toxins. When the insects are attacked by these factors, the detoxification enzymes act by regulating the metabolism of hormones, pheromones, and other biologically active substances. General esterase (EST) and glutathione *S-*transferase (GST) are the most common enzymes involved in the detoxification of penetrated xenobiotics (Zibaee et al. 2009b). In past studies, the insect resistance and activity of insect EST and GST induced by certain pesticides have been reported (Terriere 1984; Bakanova et al. 1996; Feyereisen 1999; Serebrov et al. 2006; Zibaee et al. 2009a; Zibaee et al., 2009b). However, reports are scarce on the changes in the activity of detoxification enzymes when the insects are infected by entomopathogenic fungi and/or the fungal secondary metabolites.

It is known that the entomopathogenic fungal attack of host insects involves both a direct infection by the fungus and the action of its secondary metabolites. Because some secondary metabolites are toxic to insects (Sokolova et al. 1999; Xia et al. 2000; Xia et al. 2001), both the entomopathogenic fungi and their secondary metabolites can induce immunoreactions and changes in the activity of detoxification enzymes in the host. Nevertheless, no data are available on the action and changes in the activity of the detoxifying enzymes of pine caterpillars when infected by pathogenic fungi and/or exposed to the fungal secondary metabolites.

In a previous study, a new strain, *B. brongniartii*, was isolated from the naturally diseased corpses of *D. tabulaeformis* Tsai et Liu in a pine forest at Chengde, Hebei, China; the fungal secondary metabolites comprised 2-piperridinone, 2-coumaranone, pyrrolo, and certain other toxic components (Fan et al. 2008).

In the present study, the entomopathogenic fungus *B. brongniartii* and the fungal secondary metabolites were employed as pathogens for the pine caterpillar *D. tabulaeformis*, the most destructive pest in the pine forests of northern China. The objective was to determine the changes in the GST and EST activities when the *D. tabulaeformis* larvae were infected with conidial suspensions or were exposed to the secondary metabolites of *B. brongniartii*. This study provides evidence for an understanding of the infection mechanism, the sites of fungal attack, and the immune response of the host insect.

## Material and Methods

### Insect

The larvae of *D. tabulaeformis were* collected in a pine forest in Chengde (E 117° 51′, N 40° 57′), Hebei Province, China. The larvae were reared with fresh pine needles of *Pinus tabulaeformis* Carr. in a rearing room at 27 ± 1° C, with 75 ± 10% RH, and a 15:9 L:D photoperiod. After completing two generations, the healthy fourth-instar larvae were used for the experiments.

### Entomopathogenic fungus

A *B. brongniartii* strain of entomopathogenic fungus was employed in the experiment. We isolated this strain in 2008 from the naturally infected dead larvae of *D. tabulaeformis* collected in a pine forest in Chengde. Before the experiment, the strain was cultured on potatodextrose-agar medium for 15 days at 25 ± 1° C, with 75 ± 10% RH.

### Fungal suspension preparation

After culture for 15 days, the fungal conidia were harvested from the surface of the culture medium using a sterile blade. After preparing the conidial suspensions, the conidial concentration was determined using a hemocytometer and was adjusted to 6 × 10^5^, 6 × 10^6^, and 6 × 10^7^ spores/mL with 0.1% (v/v) Tween-80 (Kermel, www.chemreagent.com) sterile water solution.

### Fungal metabolite preparation

Fungal liquid culture. The harvested conidia were prepared as suspension with a concentration of 1 × 10^8^ spores/mL. One mL aliquot of the conidial suspension was used to inoculate 100 mL liquid medium, which was prepared with 10 g/L peptone, 10 g/L yeast extract, and 10 g/L glucose in a 250 mL conical flask. The fungus was cultured in an incubator (MAXQ 5000, Thermo Scientific, www.thermoscientific.com) at a constant temperature of 25 ± 1° C and at 265 rpm for 7 days.

Extraction of fungal metabolites. After culturing, crude extracts of the cultured broth were obtained following the method reported by Hu (2006). The fermentation broth was centrifuged (Centrifuge 58042, Eppendorf, www.eppendorf.com) at 10,000 × g for 15 min and then concentrated to 1/5 at 50° C. The concentrated broth was then precipitated with alcohol (final concentration into 70% v/v) (Kermel) for 24 hr. After centrifugation (Centrifuge 58042) at 5,000 × g for 20 min, the supernatant was sequentially extracted with ethyl acetate (Kermel) at a 1:2 ratio. Lastly, an orange-red powder was obtained after drying at 40° C. This orange-red powder was considered the fungal secondary metabolites used in the experiment.

### Bioassays

Using a micro injector (Angle, www.shweiliang.com), the larvae were injected with 5 µl of a range of fungal spore concentrations (6 × 10^5^–10^7^ spores/mL) and 1 µl of the supernatant extract concentrations (5.5–550 µg/mL). Sixty samples of the larvae were treated separately for each experimental group, and an additional 60 samples of the larvae were synchronously treated with 0.1% (v/v) Tween-80 and/or DMSO (Sigma Aldrich, www.sigmaaldrich.com) (0.5%) alone as the controls. All of the experiments were conducted twice, with five replicates each. The inoculated insects were transferred to rearing chambers (SPX-2051-C, Boxun, www.boxun.com.cn) and cultured for 6 days at 25 ± 1° C with 75 ± 10% RH and a photoperiod of 15:9 L:D.

### Insect mortality

The mortality of the insects was recorded on the 7^th^ day post-treatment. The medium lethal concentration (LC_50_) was calculated using Probit Analysis of Regression (SPSS 13.0, www-01.ibm.com/software/analytics/spss).

### Enzymatic sample preparation

During culturing, the insects in each test group that were inoculated with the conidial suspensions were sampled randomly and daily from the 1^st^ day to the 6^th^ day after the treatment. The metabolite treated insects were sampled randomly at 2, 4, 6, 12, 24, and 48 hr, respectively. The sample number of the insects was 5 insects from each test group at every time point. The insect samples were homogenized using an electric homogenizer (DY89-II, Scientz, www.scientz.com) at 4° C and 690 × g for 1.5 min. Next, they were cooled in 0.1 mol/L phosphate buffer (PBS, pH 7.5) with 0.3% Triton X-100 (Solarbio, www.solarbio.cn) (tissue: PBS = 1 g:10 mL). The tissue homogenate was then centrifuged (Centrifuge 58042) for 30 min at 15,000 × g and at 4° C. The supernatant, as the enzyme source, was transferred to new tubes and stored (BCD 212DC, Haier, www.haier.com) at -20° C for 2 days only. The tests were conducted twice, with three replicates each, and the enzymatic activities associated with the observed insect resistance were determined.

### GST activity determination

The method reported by Oppenorth (1985) was adopted for determining the changes in the GST activity in the test insects; 1-chloro-2, 4-dinitrobenzene was purchased from Sigma Aldrich Company. 195 µL 1-chloro-2, 4-dinitrobenzene (20 mM) were placed in microplate wells, and 5 µl of enzyme solution was added. The optical density value at 340 nm was recorded using a microplate reader (M5, Spectra Max, www.spectramax.com).

### EST activity determination

The EST activity of the insects was determined following the method described by Han et al. (1998); α-naphthyl and β-naphthyl were purchased from Sigma Aldrich. A 135 µl aliquot of α-naphthyl acetate (or β-naphthyl) (0.3 mM) and 50 µl fast blue RR salt (Sigma Aldrich) (1 mM) were added to each well of a microplate (Costar 3590, Corning, www.corning.com). The reaction was initiated by the addition of 15 µl of enzyme solution, and the optical density value at 450 nm (M5, Spectra Max) was recorded.

### Protein determination

The protein concentrations of the enzyme solution were measured according to the method of Bradford (1976) using bovine serum albumin (Bio-Rad, www.bio-rad.com) as the Standard.

**Figure 1.**
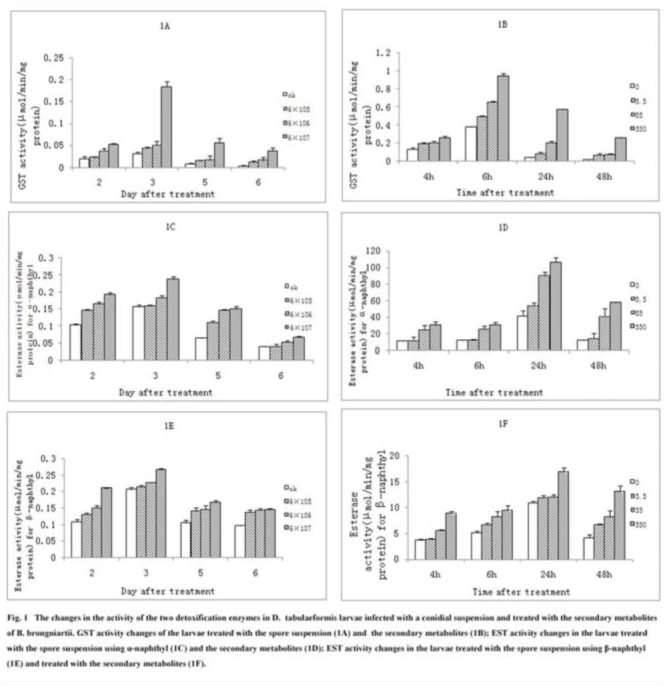
The changes in the activity of the two detoxification enzymes *Dendrolimus tabulaeformis* larvae infected with conidial suspension and treated with secondary metabolite of the fungus. (1A) GST activity changes of the larvae treated by the spore suspension; (1B) GST activity changes in larvae treated by the secondary metabolite; (1C) EST activity changes measured under the condition using α-naphtyl in the larvae treated by the spore suspension; (1D) EST activity by using α-naphtyl in larvae treated by secondary metabolite; (1E) EST activity changes measured in the condition using β-naphtyl in the larvae treated by the spore suspension; (1F) Activity of EST by using β-naphtyl in larvae treated by secondary metabolite. High quality figures are available online.

### Statistical analysis

The data were compared using the univariate analysis of the General Linear Model when significant differences were found *at p* = 0.05 (SPSS 13.0). The differences between samplings were considered statistically significant at a probability of > 5%.

## Results

### Mortality of larvae infected with the fungal conidia

After inoculation with the conidial suspension of the fungus *B. brongniartii*, the dead larvae were recorded. The mortality data are shown in Table 1. The larval mortality rate was positively correlated with the conidial concentration in the suspensions. The highest mortality of larvae was 59.67 ± 3.07% and was found in the group treated with the highest conidial concentration (6.0 × 10^7^ spores/mL). The larval mortalities were 37.00 ± 2.40% and 23.33 ± 1.73% in the groups treated with 6.0 × 10^6^ spores/mL and 6.0 × 10^5^ spores/mL, respectively. The differences in the larval mortality were significant among the groups. The LC_50_ was 2.7 × 10^7^ spores/mL (95% confidence interval).

### Mortality of larvae treated with the fungal secondary metabolites

The mortality rate of the larvae in the bioassay also positively changed with the concentration of the fungal secondary metabolites (Table 2). In the groups treated with fungal secondary metabolites at three concentrations, 5.5, 55, and 550 µg /mL, the larval mortalities were 29.33 ± 3.2%, 46.00 ± 1.47%, and 71.33 ± 3.07%, respectively. The differences were significant between the groups. The LC_50_ was 2.80 × 10^2^ µg/mL (95% confidence interval).

### GST activity alteration of larvae infected with the conidial suspension

The experimental results showed an immunoreaction in the larvae of *D. tabulaeformis* after infection with the conidial suspensions of *B. brongniartii*. The GST activity in the larvae increased from a low level to high level and then decreased again (Figure 1A). The GST activity remained at a very low level at 1 day post-infection. However, at 2 days post-infection, the GST activity increased notably and stayed high until 3 days post-infection. The difference was significant between at 3 days and at the other time points. Afterward, the enzymatic activity declined quickly, and the activity level dropped to the lowest point at 6 days, which was almost the same as that in the control. Although the GST activity of the insects fluctuated over time, the effect of the conidial concentration in the fungal suspension was marked. For example, in the 6 × 10^5^ spores/mL treatment, there were no significant differences in the GST activities between the control from 1–6 days. A significant difference (*p* < 0.05) was observed in the group treated with 6 × 10^6^ spores/mL. Higher GST activities in the host insect were also found from 1–6 days post-infection in the group infected with 6 × 10^7^ spores/mL, and the difference reached a significant level (*p* < 0.05) compared to the control. Therefore, a higher conidia concentration resulted in the induction of a higher GST activity.

However, it should be noted that with 6 × 10^7^ conidia/mL, the GST activity of the insect reached 0.184 ± 0.013 (µmol/min/mg protein) at 3 days, which was the highest value and much higher than other values (Table 3).

### EST activity alteration of larvae infected with the conidial suspension

A notable change was also found in the EST activity in *D. tabulaeformis* when the larvae were inoculated with the spore suspension of *B. brongniartii*. The EST activity was measured each day for 1–6 days post-inoculation using α-naphthyl. The results showed that the EST activity fluctuated over time. It initially began to increase, was maintained for 3 days, and then declined at 4 days (Figure 1C). The highest activity was at 3 days and was significantly different from other days (*p* < 0.05). The EST activity was positively correlated with the conidial concentration in the fungal suspension. The larvae were treated with the three concentrations of fungal suspension exhibited higher EST activities that were significant (*p* < 0.05). For example, at 3 days the EST activities were 0.156 ± 0.007 (µmol/min/mg protein) in the control, 0.159 ± 0.003 µmol/min/mg protein) in the 6 × 10^5^ spores/mL group, 0.184 ± 0.005 (µmol/min/mg protein) in the 6 × 10^6^ spores/mL group, and 0.237 ± 0.009 (µmol/min/mg protein) in the 6 × 10^7^ spores/mL group (Table 4).

A similar trend in the insect EST activity was observed using β-naphthyl in the experiment (Figure 1E), with a slight increase of the EST activity on the 1^st^ day post-inoculation, which increased for 2 days. The highest EST activity was on the 3^rd^ day; the EST activity started to decline at 4 days, and continued to decrease to the 6^th^ day. The differences between the time points were significant (*p* < 0.05). There was a notable effect of the spore concentration, the EST activities of the treated insects were all higher than in the control section, and the differences were significant (*p* < 0.05). For example, at 3 days the EST activities were 0.207 ± 0.006 (µmol/min/mg protein) in the control, 0.214 ± 0.006 (µmol/min/mg protein) in the 6 × 10^5^ spores/mL group, 0.227 ± 0.001 (µmol/min/mg protein) in the 6 × 10^6^ spores/mL group and 0.266 ± 0.005 (µmol/min/mg protein) in the 6 × 10^7^ spores/mL group (Table 5).

### GST activity alteration of larvae treated with fungal secondary metabolites

The fungal secondary metabolites altered the GST activity in the *D. tabulaeformis* larvae. Figure 1B shows that GST activity in the treated larvae fluctuted from a low to high level, and then decreased again. The highest activity of GST appeared at 6 hr, and significant differences existed at 2, 4, 6, 12, 24, and 48 hr (*p* < 0.05). The concentration of the secondary metabolite had a notable effect on the GST activity. The high dose (550 µg/mL) group displayed the highest GST activities from 2–48 hr, whereas the activities were the lowest at 5.5 µg/ml and moderate at 55 µg /mL (Table 6). The differences in the three groups were significant (*p* < 0.05). These results indicated that the secondary metabolites of the pathogenic fungus were the reason for the changes in the insect GST activity.

### EST activity alteration of larvae treated with fungal secondary metabolites

Similarly, the secondary metabolites of *B. brongniartii* induced a significant change in the EST activity of the *D. tabulaeformis* larvae. Using α-naphthyl, the EST activity displayed a stable increase during the first period of 2–24 hr post-treatment in all of the test groups, and its peak values appeared at 24 hr. After that, the EST activity declined until 48 hr. The differences in the values for the different time points were significant (*p* < 0.05). The effect of the secondary metabolite concentration in inducing the insect EST activity was marked. For example, at 24 hr the EST activity values were 54.31 ± 3.20, 90.42 ± 4.33, and 106.98 ± 5.32 (µmol/min/mg protein) for the concentrations of 5.5, 55, and 550 µg/mL, respectively; their differences were significant (*p* < 0.05) (Table 7).

Similar results were obtained using β-naphthyl. The insect EST activity increased from 2 hr to 24 hr post-treatment and declined thereafter (Figure 1F). The differences of the insect EST activities between the time points (2–48 hr) were significant (*p* < 0.05). The concentration effect of the secondary metabolite was also notable. For example, at 24 hr the values of the EST activity were 11.96 ± 0.42, 12.05 ± 0.47, and 16.98 ± 0.74 (µmol/min/mg protein), for 5.5, 55, and 550 µg/mL, respectively, with the differences being significant (*p* < 0.05) (Table 8).

The EST activity measured in the experiment using α-naphthyl was ten times as high as that measured in the experiment using β-naphthyl. The EST activity of the *D. tabulaeformis* larvae was stimulated by the fungal metabolites and correlated with the concentration.

## Discussion

Entomopathogenic fungi have been studied and applied as important biocontrol agents for certain insect pests. In contrast to the effects of fungal conidia (Lopes et al. 2011; Sabry et al. 2011; Khalid et al. 2012; Nussenbaum et al. 2012), researchers have paid more attention to the role of fungal secondary metabolites on host insects in recent years (Sokolova et al. 1999; Xia et al. 2000; Xia et al. 2001; Zibaee et al. 2009a; Zibaee et al. 2011). Abdul-Wahid et al. (2012) reported the pathogenicity of both a spore suspension and metabolite solution of two fungi, *Trichoderma harzianum* and *Fusarium solani*, against cockroaches (*Periplaneta americana*). Our study showed that *B. brongniartii* and its metabolites had toxic effects on the pine caterpillar *D. tabulaeformis* larvae, which is in agreement with previous studies. However, in comparison with the conidial infection, it was found that the fungal metabolites were more effective in killing the insects. Moreover, the time until larval mortality was shorter with the metabolites than infection with the conidia. The possible reason was that the conidial infection required more time to complete the series of infection processes, including conidial attachment on the host cuticle, germination, hyphal penetration through the integument, and infection of the internal tissues and organs, whereas the secondary metabolite attack was more direct. Hence, fungal secondary metabolites should be considered for pest management programs in the future.

It is known that certain secondary metabolites of entomopathogenic fungi, including destruxins, cylosporines, and beauverolides, possess functions in impairing the immune response of the host insect, causing death (Gillespie and Claydon 1989; Vilcinskas et al. 1997). Zibaee et al. (2011) suggested that the secondary metabolites produced by *B. bassiana* disable several immune mechanisms of *Eurygaster integriceps*, thus helping the fungus to overcome and kill its host. As the enzymatic defense in insects, detoxification enzymes play significant roles in eliminating exotic compounds and maintaining normal physiological functions (Li et al. 2007). During the degradation or sequestration of toxic compounds, the important detoxification enzymes include general ESTs, carboxylesterase, acid phosphatase, alkaline phosphatase, cytochrome P450 monooxygenases, and GSTs (Gordon 1961; Yang et al. 2001). ESTs and GSTs have been reported to be the most important detoxification enzymes that protect insects against the attack from spores and metabolites of entomopathogenic fungi. It is reported that GSTs act in the detoxification process of some insecticides (Flores et al. 2006; Gunasekaran et al. 2011; Sukhirun et al. 2011), whereas ESTs function as hydrolytic enzymes, playing a role in the cleavage of the esters that comprise organic, mineral acids and alcohols or phenols (Michèle et al. 2000). Our study showed that the activities of GST and EST were notably increased when *D. tabulaeformis* larvae were treated with a spore suspension or fungal metabolites. The defense system of the *D. tabulaeformis* larvae reacted to the attack from the conidial infection and the fungal metabolites by resisting oxidative damage and relieving oxidative stress from the toxicants and accelerating toxin metabolism. The trends of GST and EST activities were similar when the larvae were treated with the spores and metabolites. However, the time required for the alterations was different for the conidial infection and metabolite treatment (Figure 1). This result indicated that the immune reaction of the *D. tabulaeformis* larvae treated with the fungal metabolite was faster than in those infected with the fungal spores, a result different from a previous study. Zibaee et al. (2009a) reported that the time required to alter the GST and EST activities of *E. integriceps* adults infected with *B. bassiana* spores was similar to that of adults treated with extracted secondary metabolites of the fungus. This difference was due to the different host insects and the different toxin compositions from different fungi.

The detoxification enzymes of insects have many functions in repairing physiological processes, detoxifying pathogenic products, and metabolizing biologically active compounds. Hence, alterations in their activity may have an impact on the insect to adapt to the environment (Serebrov et al. 2001, 2006). Our study showed that the effect on the detoxification enzyme (EST and GST) activity in *D. tabulaeformis* larvae was closely related to the concentration of the conidia and the metabolites of *B. brongniartii*, and also to the post-treatment time.

In general, our study provides new evidence for understanding the infection mechanism of entomopathogenic fungi and the immune response of the host insect. Our results are significant for applying *B. brongniartii* and its metabolite to control the pest *D. tabulaeformis*.

**Table 1.**
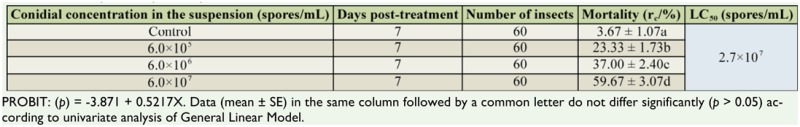
Toxicity of the spore suspension on the larvae of *Dendrolimus tabulaeformis*.

**Table 2.**
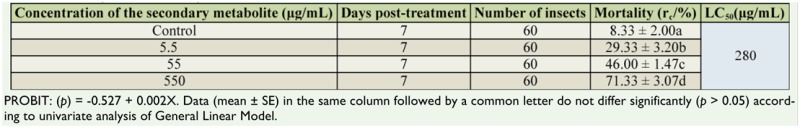
Toxicity of the secondary metabolites on the larvae *Dendrolimus tabulaeformis*.

**Table 3.**
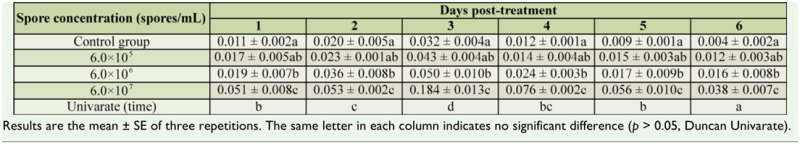
The GST activity (µmol/min/mg protein) in the *Dendrolimus tabulaeformis* larvae infected with the conidial suspension.

**Table 4.**
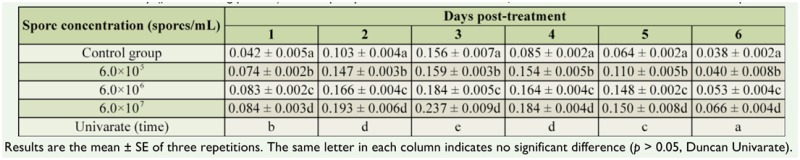
The EST activity (µmol/min/mg protein) for α-naphthyl in the *Dendrolimus tabulaeformis* larvae infected with the conidial suspension.

**Table 5.**
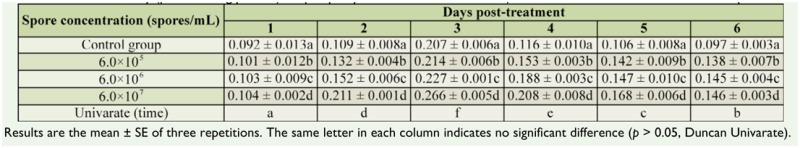
The EST activity (µmol/min/mg protein) for β-naphthyl in the *Dendrolimus tabulaeformis* larvae infected with the conidial suspension.

**Table 6.**
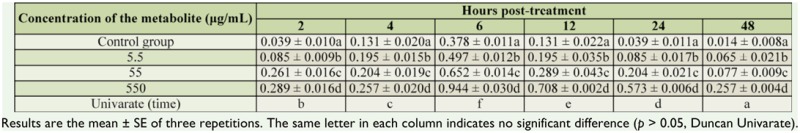
The GST activity (µmol/min/mg protein) in the *Dendrolimus tabulaeformis* larvae treated with the secondary metabolites.

**Table 7.**
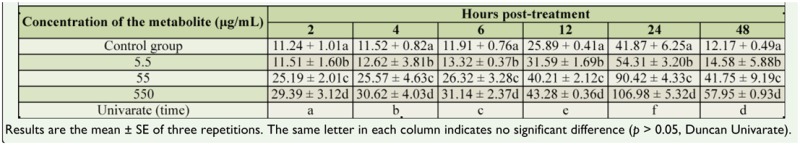
The EST activity (µmol/min/mg protein) for α-naphthyl in the *Dendrolimus tabulaeformis* larvae treated with the secondary metabolites.

**Table 8.**
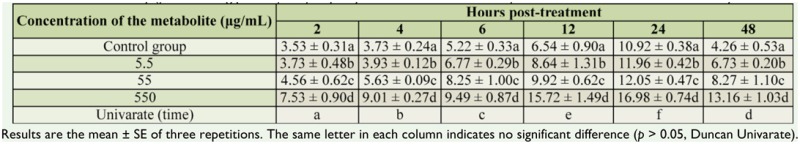
The EST activity (µmol/min/mg protein) for β-naphthyl in the *Dendrolimus tabulaeformis* larvae treated with the secondary metabolites

## Abbreviations

EST: esterase
GST: glutathione *S*-transferase
LC_50_: medium lethal concentration
